# *Escherichia coli JNL-EC1* enhances type I IFN-mediated antiviral response during DNA and RNA virus infection

**DOI:** 10.3389/fmicb.2026.1820773

**Published:** 2026-06-03

**Authors:** Rui Li, Zhaoyi Pan, Na Wang, Shujuan Zhang, Xianbo Geng, Yating Yu, Jin Shi, Cunfei Liu, Changzhong Jin, Liang Shang, Haibo Wu, Nanping Wu

**Affiliations:** 1 Shandong First Medical University and Shandong Academy of Medical Sciences, Jinan, China; 2 Jinan Microecological Biomedicine Shandong Laboratory, Jinan, China; 3 Department of Cardiac Surgery, Linyi People’s Hospital, Shandong Second Medical University, Linyi, China; 4 Department of Cardiology, Linyi People's Hospital, Shandong Second Medical University, Linyi, China; 5 State Key Laboratory for Diagnosis and Treatment of Infectious Diseases, National Clinical Research Center for Infectious Diseases, Collaborative Innovation Center for Diagnosis and Treatment of Infectious Diseases, The First Affiliated Hospital, Zhejiang University School of Medicine, Hangzhou, China; 6 Department of Gastrointestinal Surgery, Shandong Provincial Hospital Affiliated to Shandong First Medical University, Jinan, China

**Keywords:** cGAS-STING, *Escherichia*, innate immunity, intestinal bacteria, type-1 interferons

## Abstract

**Introduction:**

The gut microbiota plays a critical role in shaping host antiviral immunity, particularly through the regulation of type I interferon (IFN-I) signaling. Infants are highly vulnerable to viral infections, largely due to their adaptive immune immaturity. Identifying specific commensal bacteria capable of enhancing IFN-I responses represents a promising strategy to boost antiviral defense in this vulnerable population.

**Methods:**

Forty-five bacterial strains were isolated from fecal samples of infants aged 0-3 years. Their ability to modulate virus-induced IFN-I expression was evaluated using qPCR-based assays in cell lines infected with Sendai virus (SeV). One strain, *Escherichia coli JNL-EC1*, was selected for further genomic analysis, virulence gene profiling, and hemolysis testing. We assessed IFN-I and ISGs expression, as well as the phosphorylation of STING, TBK1, and IRF3, under conditions of cGAS-STING activation, TBK1 or IRF3-5D overexpression, and HSV-1 or SeV infection. The active components of its metabolites were fractionated using ethanol and chloroform extraction. Antiviral effects were evaluated in HEK293T, INT407, and THP-1 cells, as well as in a SeV-infected mouse model.

**Results:**

Among the 45 isolates, metabolites of *E. coli JNL-EC1* significantly enhanced SeV- and HSV-1(herpes simplex virus 1)-induced IFN-I signaling activation. Genomic analysis confirmed that *E. coli JNL-EC1* resembles a commensal strain, lacking typical pathogenic determinants and exhibiting a non-hemolytic phenotype. The metabolites potentiated cGAS/STING- and TBK1-mediated IFN-I signaling but did not further enhance IRF3-5D-induced gene expression. Moreover, the metabolites increased the phosphorylation of STING, TBK1, and IRF3 following HSV-1 or SeV infection. Active components were present in both water-soluble (0-60% ethanol) and chloroform-extracted fractions and were heat-stable, suggesting the involvement of multiple non-proteinaceous metabolites. Functionally, metabolite pretreatment reduced the replication of GFP-tagged HSV-1 and vesicular stomatitis virus (VSV) in vitro. *E. coli JNL-EC1* significantly upregulated Ifnb1 expression in the spleen and modulated both the composition and activation status of immune cell populations.

**Discussion:**

These findings identify *E. coli JNL-EC1* as a gut commensal with broad antiviral-enhancing properties and reveal a microbiota-mediated mechanism that primes antiviral immunity through the IFN-I pathway.

## 1 Introduction

The intestinal microbiota is increasingly recognized as an “invisible organ” that modulates diverse physiological processes through multiple “gut-organ axes” (Yin et al., 2024). Accumulating evidence has revealed a bidirectional regulatory relationship between viral infections and the gut microbiota (Upadhyay et al., 2023; Brown et al., 2022; Nagata et al., 2023). As a key component of the gut microbial community, commensal bacteria play an essential role in shaping antiviral immune responses (Stefan et al., 2020). Innate immunity serves as the first line of defense against pathogenic viruses, mediating both viral sensing and restriction. A recent study demonstrated that commensal bacteria regulate antiviral immunity via a bile acid-type I interferon (IFN-I) signaling axis (Winkler et al., 2020). Moreover, alterations in the composition of the gut microbiota can influence host antiviral responses by modulating MAVS- or STING-mediated interferon signaling pathways (Wang et al., 2023). In recent years, growing attention has focused on defining the precise mechanisms by which specific commensal bacteria govern host immune defense against viral infections. For instance, the acetate-producing bacterium *Bifidobacterium pseudolongum NjM1* has been shown to promote IFN-I production through the acetate-GPR43-NLRP3-MAVS-IFN-I signaling cascade (Niu et al., 2023).

Following infection with pathogenic viruses, pattern recognition receptors (PRRs)-including Toll-like receptors (TLRs), retinoic acid-inducible gene I (RIG-I)-like receptors (RLRs), NOD-like receptors (NLRs), and cyclic GMP-AMP synthase (cGAS)-recognize viral nucleic acids and initiate signaling cascades that culminate in the production of proinflammatory cytokines and IFN-I (Gong et al., 2020; Isazadeh et al., 2023; Chan and Gack, 2016). Upon RNA virus infection, viral RNA is recognized by cytosolic RLRs, which signal through the adaptor protein MAVS to activate TBK1. TBK1 subsequently phosphorylates the transcription factor IRF3, thereby inducing IFN-I expression and antiviral responses (Broquet et al., 2011). In the case of DNA virus infection, viral DNA is sensed by the cytosolic DNA receptor cGAS, which utilizes ATP and GTP to synthesize the second messenger 2′3′-cGAMP, a potent agonist of the stimulator of interferon genes (STING). Binding of 2′3′-cGAMP to ER-localized STING triggers its translocation from the ER membrane through the Golgi apparatus to post-Golgi compartments. At these sites, STING recruits and activates the kinase TBK1, which in turn phosphorylates IRF3 (Pan et al., 2026). The activation and resolution of these pathways are tightly regulated, and dysregulation of these processes is frequently linked to disease pathogenesis (Shmuel-Galia et al., 2021; Kulkarni et al., 2024). The majority of the human microbiota resides within the gastrointestinal tract, where this complex microbial community plays a pivotal role in modulating host immune responses through diverse host–microbe interactions (Wang et al., 2024; Park et al., 2023). Nevertheless, only a limited subset of intestinal microbes have thus far been identified as possessing immunomodulatory functions during viral infections.

Previous studies have demonstrated that gut microbiota can enhance IFN-I expression and potentiate antiviral responses against both RNA and DNA viruses through their metabolites (Wang et al., 2023; Niu et al., 2023). These findings support the feasibility of boosting antiviral immunity via gut microbiota modulation. Infants and young children are particularly vulnerable to viral infections due to their immature immune systems, particularly adaptive immunity. Furthermore, given that their hepatic and renal functions are not yet fully developed for drug metabolism, many antiviral medications are not suitable for use in this population. Therefore, it is of particular importance to isolate and screen gut microbiota from infant feces that are capable of promoting IFN-I expression and exerting antiviral effects. Applying these microbiota to infants and young children may represent a promising strategy to enhance their resistance to viral infections.

*Escherichia coli* is a Gram-negative, rod-shaped bacterium recognized as an opportunistic pathogen in both humans and animals, largely attributed to its ability to form biofilms (Zang et al., 2023). Previous studies have reported that *E. coli* infection can lead to diarrhea (Gaastra et al., 2014; Okuno et al., 2023) and promote hepatic inflammation and fibrosis (Xin et al., 2022). Specifically, gastric administration of *E. coli* was shown to induce TNFα expression through activation of the TLR4-mediated NF-κB signaling pathway (Ma et al., 2024), highlighting the pro-inflammatory potential of this bacterium. However, whether *E. coli* is capable of modulating antiviral immunity remains unclear. In this study, we isolated 45 strains of intestinal bacteria from fecal samples of infants aged 0–3 years. Using qPCR-based assays, we evaluated the ability of these bacteria to regulate IFN-I expression induced by viral infection. Our results demonstrate that metabolites derived from one of these isolates, *E. coli JNL-EC1*, enhance IFN-I expression in response to both RNA and DNA virus infections. Moreover, pretreatment with *E. coli JNL-EC1* suppresses the replication of RNA and DNA viruses.

## 2 Materials and methods

### 2.1 Reagents, antibodies, cells and viruses

Vancomycin (Beyotime, Y256394-5g); Neomycin sulfate (Beyotime, ST2528-100g); Metronidazole (Beyotime, Y175738-500g); Ampicillin (Beyotime, ST007); Ionomycin (Beyotime, S1672); PMA (Sigma, P1585); Brefeldin A (BioLegend, 420601); RIPA lysis buffer (Beyotime Biotechonology, P0013C); AG RNAex Pro Reagent (AG, 21102); SYBR Green (ABclonal, RK21220); ABScript Neo RT Master Mix (ABclonal, RK20433); Beta-Actin Rabbit monoclonal antibody (ABclonal, AC026); Phospho-STING Rabbit monoclonal antibody (ABclonal, AP1369); STING Rabbit monoclonal antibody (ABclonal, A21051); Phospho-TBK1 (Ser172) Rabbit monoclonal antibody (Diagbio, db13985); TBK1 Recombinant Rabbit monoclonal antibody (Diagbio, db11086); Phospho-IRF3 (Ser386) antibody (Abways, CY6575); IRF3 Rabbit monoclonal antibody (ABclonal, A19717); THP-1, INT-407, Vero, BHK-21 and HEK293T cells were purchased from ATCC. HSV-1, VSV, and SeV were stored in our own laboratory. The HSV-1 stock was prepared on Vero cells, the VSV stock on BHK-21 cells, and the SeV stock on chicken embryo cells.

### 2.2 Mice

C57BL/6 mice that were purchased from Beijing HFK Bioscience (Beijing, China) were used for all experiments. All animal experiments were carried out in Jinan Microecological Biomedicine Shandong Laboratory (Jinan, China) according to procedures approved by the institutional ethics committee (Animal testing approval number:2024007).

### 2.3 RNA extraction and qPCR

Total RNA was isolated from cells using AG RNAex Pro Reagent. After reverse-transcription with ABScript Neo RT Master Mix, the cDNA was diluted 50-fold and subjected to qPCR analysis to measure mRNA levels of the tested genes. Data shown were the relative abundance of the indicated mRNAs normalized to that of *ACTB*. Gene-specific primer sequences were as follows: *ACTB*: CACCATTGG CAATGAGCGGTTC (forward) and AGGTCTTTGCGGATGTC CACGT (reverse), *Actb*: CATTGCTGACAGGATGCAGAAGG (forward) and TGCTGGAAGGTGGACAGTGAGG (reverse), *IFNB*: GACAGGATGAACTTTGACATCCC (forward) and CTCAAC AATAGTCTCATTCCAGCC (reverse), *Ifnb*: GCCTTTGCCATCC AAGAGATGC (forward) and ACACTGTCTGCTGGTGGAGTTC (reverse), *ISG15*: CTCTGAGCATCCTGGTGAGGAA (forward) and AAGGTCAGCCAGAACAGGTCGT (reverse), *ISG54*: GGAGCA GATTCTGAGGCTTTGC (forward) and GGATGAGGCTTCCAG ACTCCAA (reverse), *ISG56*: GCCTTGCTGAAGTGTGGAGGAA (forward) and ATCCAGGCGATAGGCAGAGATC (reverse), *IL6*: AGACAGCCACTCACCTCTTCAG (forward) and TTCTGCCAGT GCCTCTTTGCTG (reverse), SeV: ATGGCCGGGTTGTTGAGC ACCTT (forward) and GTGAGCTAGGAAGGTTGTTGCAA (reverse).

### 2.4 Freeze-drying of *E. coli JNL-EC1* metabolites

A 100 μL aliquot of an *E. coli JNL-EC1* culture (OD600 = 0.6) was used to inoculate 50 mL of MRS Broth. The culture was incubated anaerobically at 37 °C for 24 h. Following incubation, the bacterial cells were pelleted by centrifugation at 12,000 × g for 10 min at room temperature. The resulting supernatant was filter-sterilized using a 0.45-μm membrane filter and subsequently lyophilized to dryness. The mass of the resulting dried residue was then recorded.

### 2.5 *Escherichia coli JNL-EC1* metabolites dissolved in either ethanol or chloroform on regulation of the IFN-I antiviral signaling pathway

Five mL of lyophilized metabolites from *E. coli JNL-EC1* were reconstituted in 1 mL of varying concentrations of ethanol and chloroform. After centrifugation at 12,000 × *g* for 2 min, the supernatants were transferred to new 1.5 mL microcentrifuge tubes, and insoluble fractions were discarded. THP-1 cells (5 × 10^5^ cells per well in a 12-well plate) were pretreated for 2 h with 10 μL of the lyophilized *E. coli JNL-EC1* metabolites reconstituted in different ethanol or chloroform concentrations, or with heat-treated (100 °C for 15 min) bacterial culture supernatant, followed by infection with HSV-1 (MOI = 1). At 24 h postinfection, cells were harvested, and qPCR was performed to measure mRNA expression levels of indicated genes.

### 2.6 *Escherichia coli JNL-EC1* isolation, identification and growth conditions

Stool samples were obtained from seven infants and young children (aged 0–3 years) with no history of illness or antibiotic use. The samples were diluted and plated onto MRS Broth agar plates, which were then incubated under anaerobic conditions (80% N_2_, 10% CO_2_, 10% H_2_) at 37 °C for 2–3 days. Single colonies were identified using MALDI-TOF mass spectrometry and 16S rRNA sequencing.

Genomic DNA was extracted using a bacterial/fungal DNA extraction kit (magnetic beads) (Majorbio, Shanghai, China) according to the manufacturer’s protocol. Whole-genome sequencing of JNL-EC1 was performed commercially by Magigene Biotechnology (Guangzhou, China) using the Illumina MiSeq platform. Raw reads were quality-checked and assembled with SPAdes v3.15.5. The quality of the *E. coli JNL-EC1* genome assembly was evaluated using QUAST v5.3.0. For phylogenetic inference, the genome sequence was submitted to the AutoMLST2 online server^1^ for automated multi-locus sequence typing and phylogenetic tree reconstruction. The resulting tree was visualized using the interactive tree of life (iTOL) online tool. The genome was annotated using Prokka v1.14.6, and the annotated genome was uploaded to ChiPlot^2^ to generate a circular genome map, displaying CDS, GC content, GC skew, and RNA genes. Virulence factors were identified using the virulence factor database (VFDB)^3^.

### 2.7 Hemolysis assay

To evaluate the hemolytic activity of *E. coli JNL-EC1*, a blood agar plate assay was performed. Overnight cultures of *E. coli JNL-EC1* were streaked onto tryptic soy agar plates supplemented with 5% (v/v) defibrinated sheep blood (Columbia blood agar). The plates were incubated anaerobically at 37 °C for 48 h and then examined for the presence of a clear zone of hemolysis (*β*-hemolysis) or greenish discoloration (*α*-hemolysis) around the bacterial colonies.

### 2.8 CCK8 assay

HEK293T, THP-1 and INT-407 cells were plated in 96-well plates at 1 × 10^4^ cells/well. Twelve hour later, different doses of *E. coli JNL-EC1* metabolites were added to the 96-well plates. Thirty-six hour later, cell culture supernatants were removed and 100 μL new DMEM medium with 10 μL CCK8 was added to each well. The culture plates were incubated at 37 °C for 1 h, and the absorbance at 450 nm was determined using a microplate reader (Agilent-BioTek, Synergy Hl).

### 2.9 Western blot

THP-1 cells were pretreated with metabolites derived from *E. coli JNL-EC1*, followed by infection with HSV-1 or SeV. After 12 h, cells were harvested by centrifugation for protein extraction. For overexpression experiments, HEK293T cells were similarly pretreated with *E. coli JNL-EC1* metabolites prior to transfection with a TBK1 overexpression plasmid. At 24 h post-transfection, cells were collected, and total protein was extracted using RIPA lysis buffer supplemented with 1 mM phenylmethylsulfonyl fluoride (PMSF). Protein samples (10 μg per lane) were separated by SDS-PAGE and transferred onto PVDF membranes. Membranes were blocked with 5% skim milk in TBS and then immunoblotted with antibodies against phospho-STING, STING, phospho-TBK1, TBK1, phospho-IRF3, IRF3, beta-actin, and GFP.

### 2.10 Cells preparation

Spleens harvested from mice were homogenized in phosphate-buffered saline (PBS) and passed through a 200-mesh sieve. The homogenate was centrifuged at 400 × g for 5 min, and the supernatant was carefully removed. Erythrocytes in the pellet were lysed by resuspending the cells in 3 mL of red blood cell lysis buffer and incubating for 10 min at room temperature. Lysis was quenched by adding 10 mL of PBS, followed by centrifugation at 400 × g for 5 min. The supernatant was discarded, and the cell pellet was resuspended in PBS. The resulting cell suspension was filtered through a 200-mesh sieve to obtain purified splenic mononuclear cells.

To isolate peripheral blood mononuclear cells (PBMCs), 0.5 mL of mouse blood was collected and transferred into a tube containing sodium heparin as an anticoagulant. Erythrocytes were lysed by adding 1 mL of red blood cell lysis buffer and incubating the mixture at 4 °C for 10 min. Following centrifugation at 400 × g for 5 min, the supernatant was carefully removed and discarded. The cell pellet was resuspended in 1 mL of phosphate-buffered saline (PBS) and subjected to another centrifugation step at 400 × *g* for 5 min. After discarding the supernatant, the cells were resuspended in PBS and passed through a 200-mesh filter to obtain a homogeneous single-cell suspension of mouse PBMCs.

### 2.11 Flow cytometry analysis

Harvested mononuclear cells were incubated with respective antibodies for 30 min at room temperature after washing twice with 1 × phosphate buffer solution (PBS). For intracellular cytokines and intracellular markers staining, the cells were pre-treated with 1 μg/mL ionomycin and 50 ng/mL PMA for 4 h in the presence of Brefeldin A for the last 4 h. All stained cells were measured using LSR Fortessa (BD, USA). The data were analyzed with FlowJo software.

### 2.12 Animal study

To establish a mouse model with depleted intestinal commensal bacteria, five- to six-week-old specific pathogen-free (SPF) C57BL/6 mice were maintained under controlled conditions (temperature: 24 ± 2 °C; humidity: 40–70%; 12-h light/dark cycle) and administered an antibiotic cocktail (0.5 g/L vancomycin, 1 g/L neomycin sulfate, 1 g/L metronidazole, and 1 g/L ampicillin) in drinking water for five consecutive days, followed by 2 days of regular water. Mice were then inoculated via gastric gavage with 10^8^ CFU of *E. coli JNL-EC1*. One week later, the mice were infected intraperitoneally with SeV. Body weight was monitored daily following *E. coli JNL-EC1* administration. At the experimental endpoint, mice were deeply anesthetized with 4% isoflurane and euthanized by cervical dislocation. Blood samples were immediately collected via the retro-orbital sinus, and spleens were harvested for subsequent cell preparation.

### 2.13 Statistical analysis

Statistical significance was calculated using an unpaired Student’s t test (two-tailed) and One-way ANOVA. The results were presented as the mean ± S. D. of at least three independent experiments, *P < 0.05; **P < 0.01; ***P < 0.001. GraphPad Prism 9.5.0 was used for statistical analysis.

## 3 Results

### 3.1 Intestinal bacteria modulate virus-induced IFN-I expression

**Figure 1:**
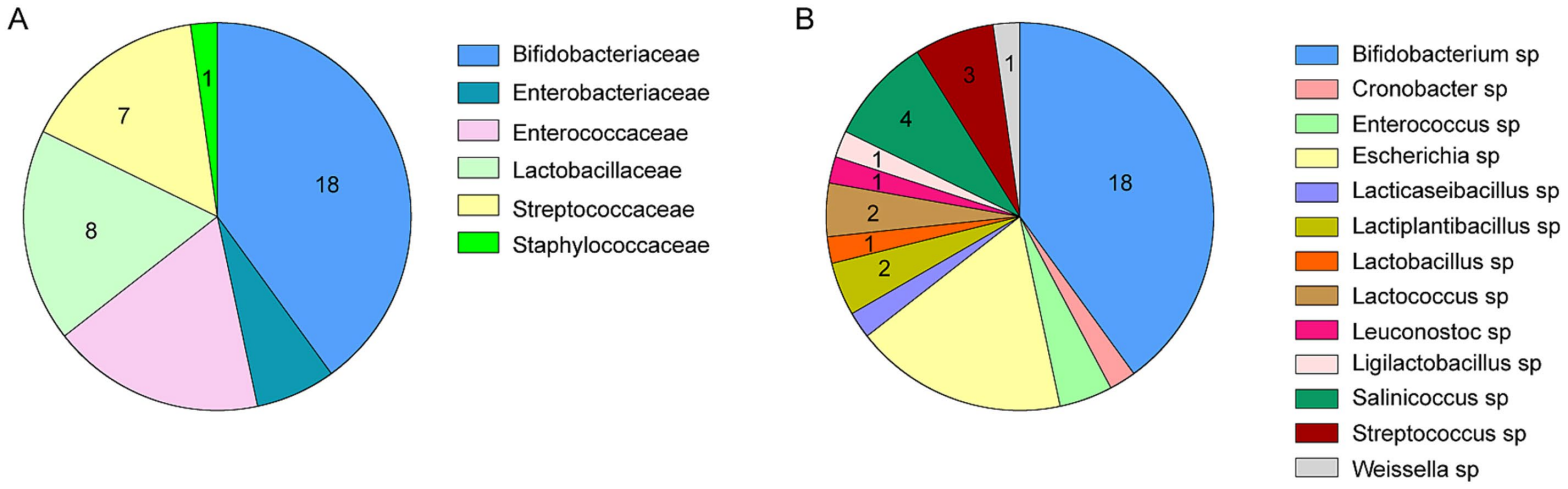
Taxonomic profiles of intestinal bacteria. **(A,B)** Pie charts represent the taxonomic distribution of intestinal microbiota at the family **(A)** and genus **(B)** levels.

To identify gut bacteria with immunomodulatory functions, we isolated 45 bacterial strains (Supplementary Table 1) from fecal samples of infants aged 0–3 years. These isolates were classified into six families-*Bifidobacteriaceae, Enterobacteriaceae, Enterococcaceae, Lactobacillaceae, Streptococcaceae,* and *Staphylococcaceae*-among which *Bifidobacteriaceae* exhibited the highest relative abundance (Figure 1A). At the genus level, the 45 strains represented 13 genera, including *Bifidobacterium, Cronobacter, Escherichia, Enterococcus, Lacticaseibacillus, Lactiplantibacillus, Lactobacillus, Leuconostoc, Ligilactobacillus, Weissella, Lactococcus, Streptococcus,* and *Salinicoccus* (Figure 1B).

### 3.2 *Escherichia coli JNL-EC1* metabolites promote *IFNB1* expression induced by SeV infection

**Figure 2:**
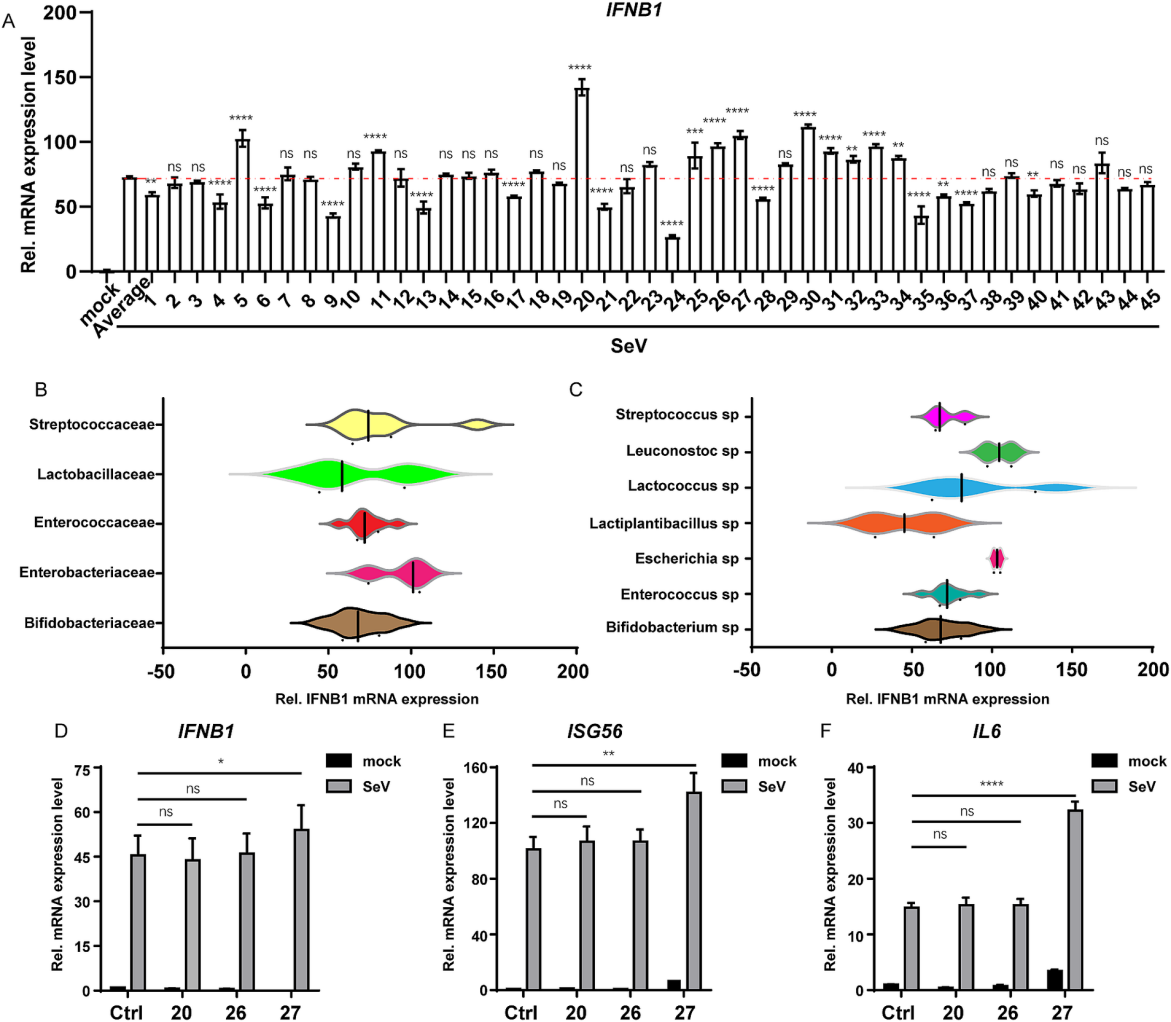
*Escherichia coli* JNL*-EC1* enhances SeV-induced antiviral responses. **(A)** THP-1 cells were pre-incubated with 45 individual bacterial metabolites (final concentration: 40 μg/mL) for 2 h prior to SeV infection. At 12 h post-infection, cells were harvested, and *IFNB1* mRNA expression was quantified by qPCR. **(B,C)** Taxonomic analysis of intestinal bacteria showing differential regulation of *IFNB1* expression by bacterial families **(B)** and genera **(C)**. **(D–F)** THP-1 cells were pretreated for 24 h with 20 μg/mL metabolites from three bacterial strains prior to SeV infection. At 12 h post-infection, cells were harvested for qPCR analysis of the *IFNB1* **(D)**, *ISG56* **(E)**, and *IL6* **(F)**. **(A)** Each group was compared with the average group. ns, not significant; *p < 0.05, **p < 0.01, ***p < 0.001, and ****p < 0.0001; Student’s *t*-test, *n* = 3.

To determine which of these commensal bacteria influence virus-induced IFN-I expression, we assessed their effects on *IFNB1* expression following Sendai virus (SeV) infection using quantitative PCR. Among the tested strains, supernatants from 13 bacterial species significantly suppressed SeV-induced IFN-I expression, whereas those from 11 species markedly enhanced it (Figure 2A). To systematically investigate the immunomodulatory potential of gut microbiota, we assessed the effects of metabolites from various intestinal bacterial strains on *IFNB1* expression based on their phylogenetic relationships (Figures 2B,C). Our findings revealed that members of the *Enterobacteriaceae* family enhanced *IFNB1* expression (Figure 2B). At the genus level, *Escherichia* and *Leuconostoc* species potentiated, while *Lactiplantibacillus* species attenuated, *IFNB1* expression following SeV infection (Figure 2C). Notably, strains 20#, 26#, and 27# significantly upregulated *IFNB1* expression (Figure 2A). Given this potent effect, we further examined whether metabolites from these strains could enhance IFN-I signaling at low doses. Among them, metabolites from strain 27# (*Escherichia coli,* designated *E. coli JNL-EC1* in our study) consistently promoted both *IFNB1* and *ISG56* expression (Figures 2D,E). Additionally, SeV infection is known to induce cytokine expression through NF-κB activation (Popli et al., 2022; Xu et al., 2019; Fang et al., 2021). We observed that metabolites from *E. coli JNL-EC1* also significantly upregulated *IL6* expression (Figure 2F), suggesting broader immunostimulatory effects beyond the IFN-I pathway.

### 3.3 Genome assembly, annotation, and phylogenetic identification of *E. coli JNL-EC1*

**Figure 3:**
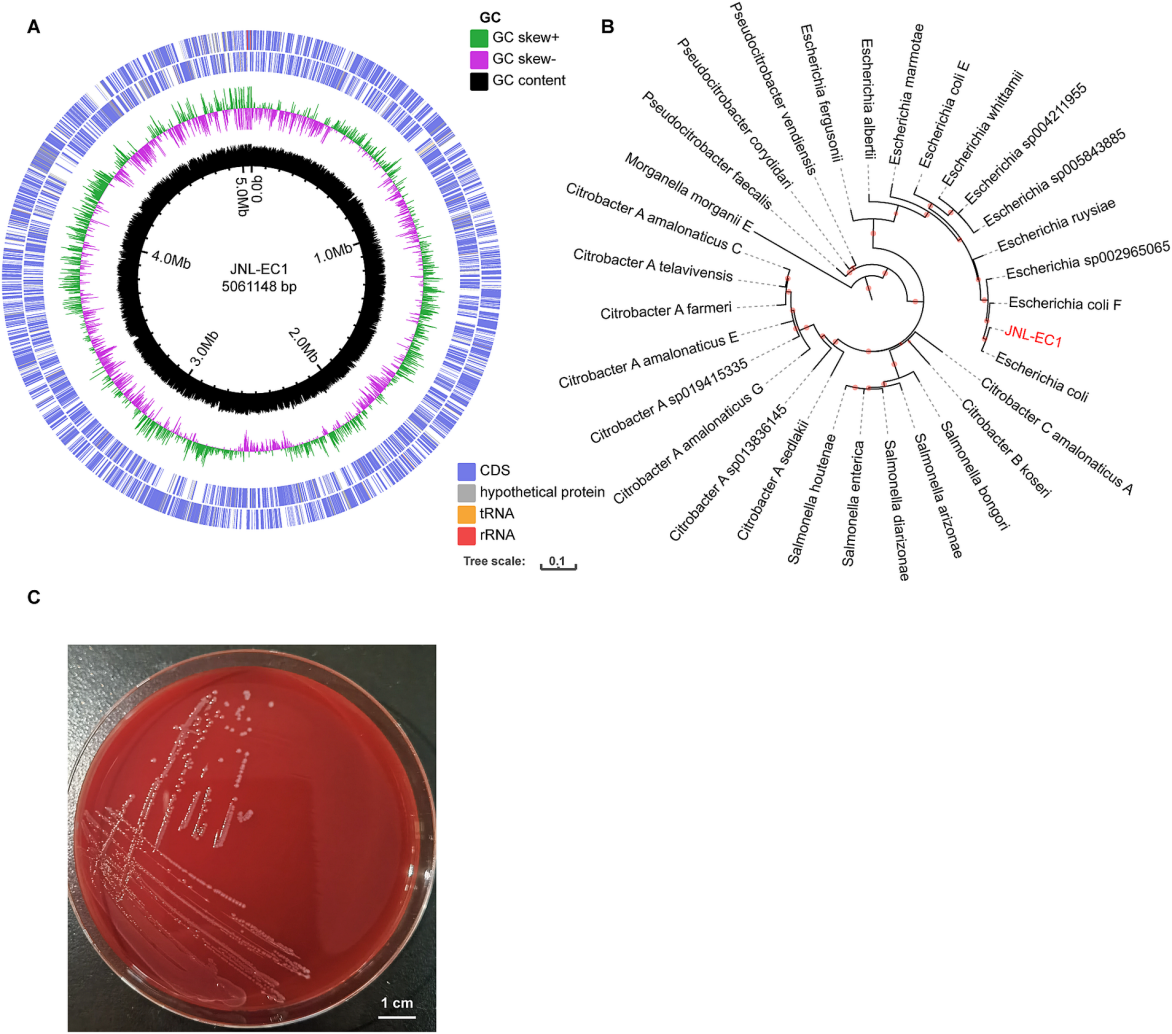
Phylogenetic tree, circular genome map, and hemolysis assay of JNL-EC1. **(A)** Phylogenetic tree of JNL-EC1 based on core genome MLST. The tree was constructed using Auto MLST2 and visualized with iTOL. JNL-EC1 is highlighted in red. Bootstrap values (>60%) are shown at nodes. The scale bar represents the number of substitutions per site. **(B)** Circular genome map of JNL-EC1. From the inner to the outer circles: genome scale (circle 1), GC content (circle 2), GC skew (circle 3), and locations of genes (CDS with known function, hypothetical protein, tRNA, and rRNA) in forward and reverse strand (circle 4 and circle 5), respectively. **(C)** Hemolytic activity of JNL-EC1.

To assess whether *E. coli JNL-EC1*, which promotes *IFNB1* expression following viral infection, harbors pathogenic genes or exhibits pathogenicity, we performed next-generation sequencing-based genomic analysis. The genome of *E. coli JNL-EC1* was assembled and annotated, revealing a total genome size of 5,061,148 bp (approximately 5.06 Mb) with a G + C content of 50.58%. A total of 5,074 protein-coding sequences (CDSs) were predicted, of which approximately 4,506 were functionally annotated (Figure 3A). To determine the taxonomic position of *E. coli JNL-EC1*, a phylogenetic tree was constructed using the AutoMLST2 online platform based on the core genome MLST scheme. As shown in Figure 3B, *E. coli JNL-EC1* clustered within the *Escherichia coli* clade with high bootstrap support, confirming its identification as a strain of *E. coli*.

The virulence gene profile of *E. coli JNL-EC1* was analyzed using the VFDB database (Supplementary Table 2). The strain harbors genes encoding ELF fimbriae (elfACDG), ECP fimbriae (ecpABCDE), type I fimbriae (fimDFGH), and several autotransporters (agn43, cah, ehaB, upaG). For iron uptake, the strain carries the heme uptake system (chuASTUWXY), but lacks the core salmochelin synthesis genes (iroB, iroC). And no genes encoding yersiniabactin, colibactin and aerobactin were found in this strain. Multiple Non-LEE encoded TTSS effectors (espL1, espL4, espR1, espX, espY) were detected; however, no LEE-encoded genes (including eae, T3SS structural genes, and LEE effectors) were found, indicating the absence of a functional T3SS injectisome. Additionally, the strain lacks potent toxins such as Shiga toxins, heat-labile/heat-stable enterotoxins, cytotoxic necrotizing factor, and cytolethal distending toxin. Only hlyE (silent hemolysin) was detected, without hlyA. Results of hemolysis assay showed no hemolytic zone was observed around *E. coli JNL-EC1* colonies, indicating a non-hemolytic (*γ*-hemolytic) phenotype (Figure 3C). Taken together, *E. coli JNL-EC1* does not carry the typical virulence determinants of pathogenic *E. coli* (e.g., EPEC, EHEC, NMEC, ETEC), and its virulence gene profile is consistent with that of a commensal or low-virulence strain.

### 3.4 *Escherichia coli JNL-EC1* metabolites enhance HSV-1-induced expression of *IFNB1* and *ISGs*

**Figure 4:**
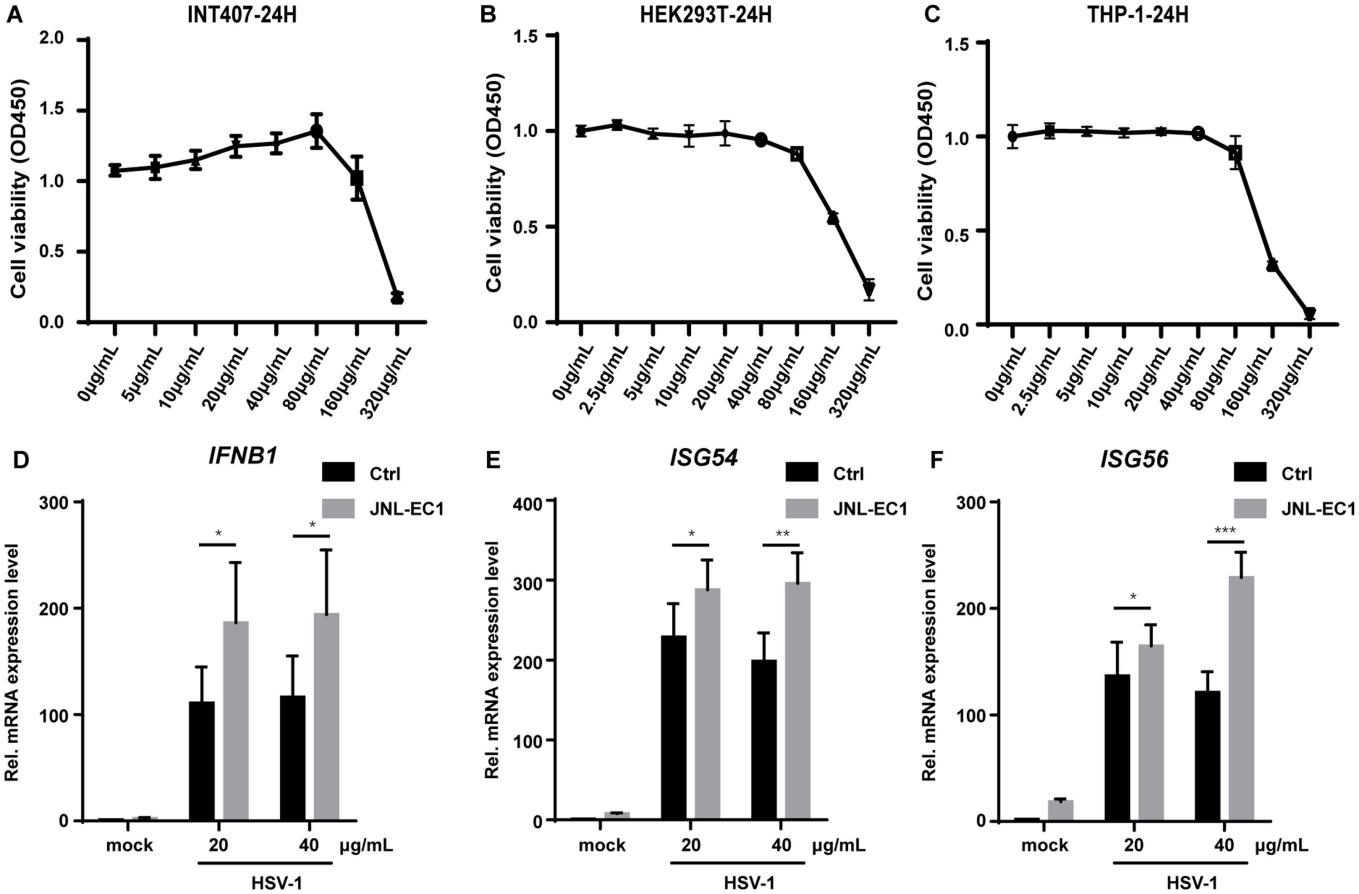
*Escherichia coli* JNL*-EC1* metabolites enhance HSV-1-induced antiviral responses. **(A–C)** Cytotoxicity assessment of *E. coli* JNL-EC1 metabolites in: INT407 **(A)**, HEK293T **(B)**, and THP-1 **(C)**. Cells were exposed to metabolite gradients (0–320 μg/mL) for 24 h before CCK-8 viability assays. **(D–F)** THP-1 cells were pretreated with 20 μg/mL and 40 μg/mL intestinal bacteria metabolites followed by HSV-1 infection. Twelve hour later, cells were collected and subjected to qPCR analysis to measure mRNA levels of the *IFNB1* **(D)**, *ISG54* **(E)**, and *ISG56* **(F)**. ns, not significant; *p < 0.05, **p < 0.01, and ***p < 0.001; Student’s *t*-test, *n* = 3.

The cytotoxic potential of metabolites derived from *E. coli JNL-EC1* was assessed in HEK293T, INT407, and THP-1 cell lines. Treatment with these metabolites at a concentration of 80 μg/mL resulted in cell viability exceeding 95% at 24 h post-treatment (Figures 4A–C), indicating minimal cytotoxicity at concentrations below this threshold. RNA viruses are known to induce IFN-I expression primarily through the activation of RIG-I-like receptors (RLRs) and their adaptor protein MAVS, as well as several Toll-like receptor (TLR) pathways (Dobrikov et al., 2023; Mantovani et al., 2023; Kato et al., 2006). In contrast, DNA viruses typically engage innate immune sensors such as cyclic GMP-AMP synthase (cGAS) to initiate interferon production (Sun et al., 2013; Hu et al., 2016). We therefore investigated whether metabolites from *E. coli JNL-EC1* could potentiate the interferon response triggered by herpes simplex virus 1 (HSV-1) infection. Our results demonstrate that pretreatment with *E. coli JNL-EC1* metabolites significantly enhances HSV-1-induced expression of *IFNB1, ISG54*, and *ISG56*. This enhancement exhibited a modest dose-dependent effect across the three genes examined (Figures 4D–F).

### 3.5 *Escherichia coli JNL-EC1* metabolites promote IFN-I production induced by cGAS/STING, TBK1

**Figure 5:**
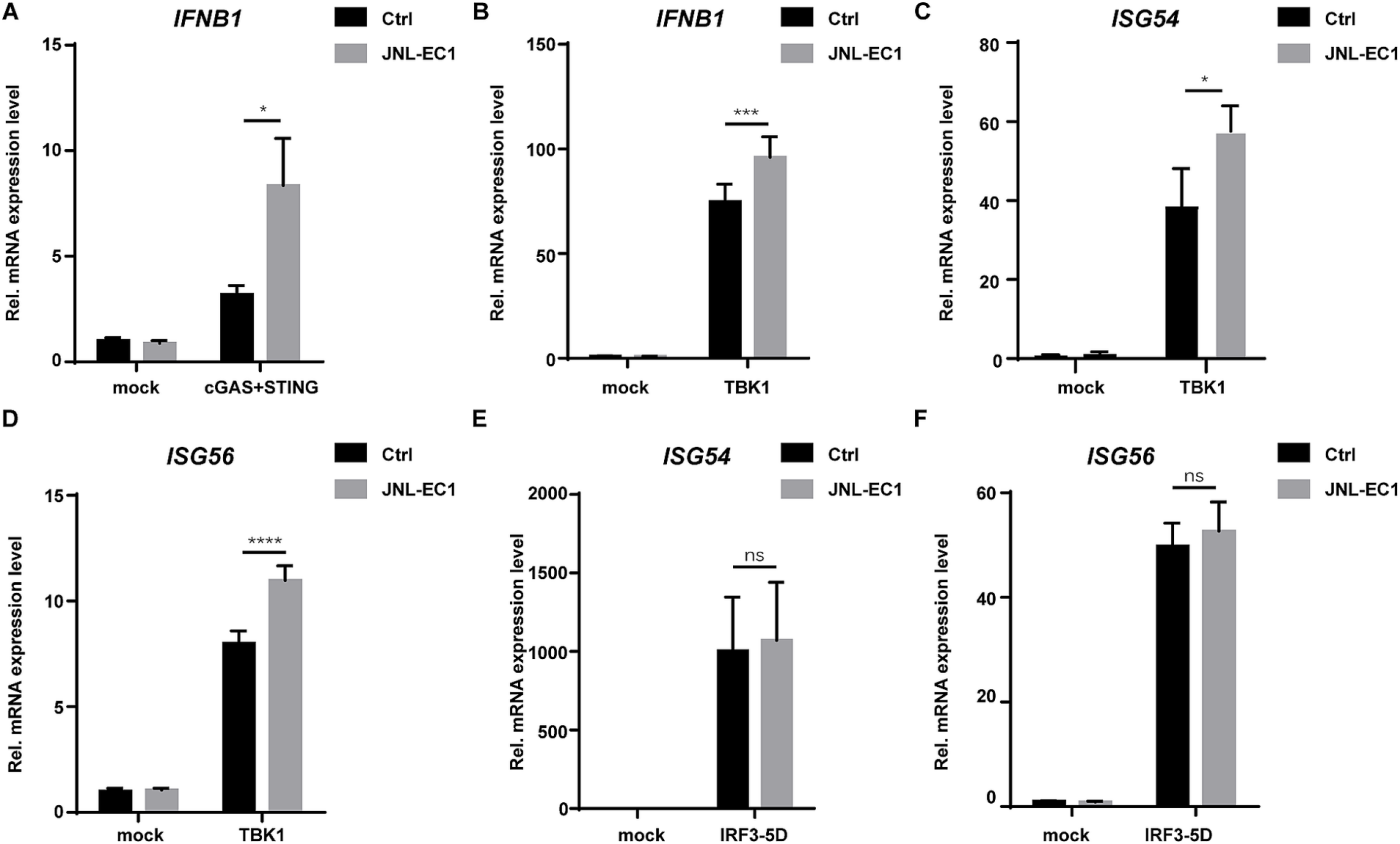
*Escherichia coli* JNL*-EC1* promote type I IFN production induced by cGAS/STING, TBK1. **(A)** HEK293T cells transfected with pcDNA3.1-cGAS/STING for 12 h were treated with 20 μg/mL *E. coli* JNL*-EC1* metabolites. After 12 h, *IFNB1* expression was quantified by qPCR. **(B–D)** Cells transfected with TBK1 overexpression plasmid were metabolite-treated as in **(A)**, followed by qPCR analysis of: *IFNB1* **(B)**, *ISG54* **(C)**, *ISG56* **(D)**. **(E,F)** IRF3-5D-transfected cells (constitutively active mutant) were analyzed for: *ISG54* **(E)** and *ISG56* **(F)**. ns, not significant; *p < 0.05, ***p < 0.001, and ****p < 0.0001; Student’s *t*-test, *n* = 3.

To further investigate the mechanism by which *E. coli JNL-EC1* metabolites enhance IFN-I production induced by viral infection, HEK293T cells were transfected with cGAS/STING, TBK1, or IRF3-5D plasmids in combination with treatment of *E. coli JNL-EC1* metabolites. Consistent with viral infection, *E. coli JNL-EC1* metabolites significantly potentiated *IFNB1* transcription induced by cGAS/STING or TBK1 overexpression (Figures 5A,B). Additionally, treatment with *E. coli JNL-EC1* metabolites positively modulated the expression of *ISG54* and *ISG56* induced by TBK1 overexpression (Figures 5C,D). However, this synergistic effect was not observed for promoting the mRNA levels of *ISG54* and *ISG56* induced by IRF3-5D overexpression (Figures 5E,F). All these results demonstrated that *E. coli JNL-EC1* metabolites could be interact with multiple targets upstream of IRF3 to enhance the IFN-I signaling pathway.

### 3.6 *Escherichia coli JNL-EC1* metabolites potentiate antiviral signaling induced by DNA and RNA viruses

**Figure 6:**
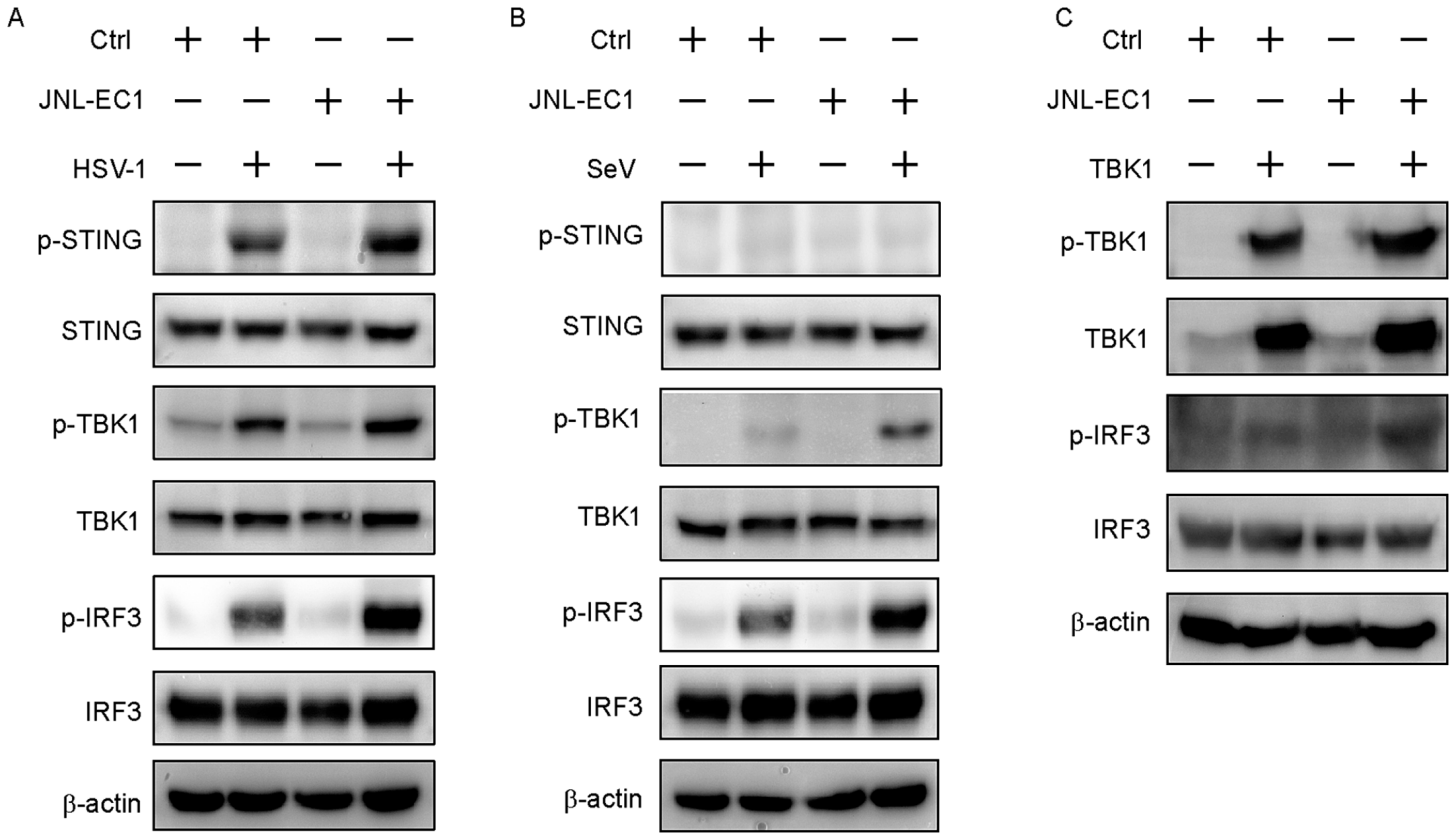
*Escherichia coli* JNL*-EC1* enhances DNA/RNA virus induced antiviral signal pathways. **(A,B)** THP-1 monocytes pretreated with 20 μg/mL JNL-EC1 metabolites for 2 h were infected with HSV-1 **(A)** /SeV **(B)**. Cells were harvested at 12 hpi for western blot analysis of antiviral signaling proteins. **(C)** HEK293T cells transfected with TBK1 overexpression plasmid for 12 h were treated with metabolites as above, followed by immunoblotting. All assays were performed in triplicate.

Phosphorylation of key adaptor proteins serves as a critical marker for the activation of antiviral signaling pathways. Upon pathogenic infection, microbial DNA is sensed by the cGAS-STING pathway, triggering downstream phosphorylation cascades involving STING, TBK1, and IRF3. Our results demonstrate that metabolites derived from *E. coli JNL-EC1* significantly enhance HSV-1-induced phosphorylation of STING, TBK1, and IRF3 (Figure 6A). In parallel, RNA viruses are detected by RLRs, which initiate IFN-I signaling. Consistent with the DNA virus results, *E. coli JNL-EC1* metabolites also potentiated TBK1 and IRF3 phosphorylation during SeV infection (Figure 6B). Given that both DNA and RNA viruses converge on TBK1/IRF3 activation, we further examined the effect of these metabolites in a TBK1-overexpression model. Notably, the metabolites facilitated TBK1 and IRF3 phosphorylation under conditions of TBK1 overexpression (Figure 6C), suggesting a broad enhancement of antiviral signaling initiation.

### 3.7 Multiple components in the metabolites of *E. coli JNL-EC1* regulate antiviral immunity

**Figure 7:**
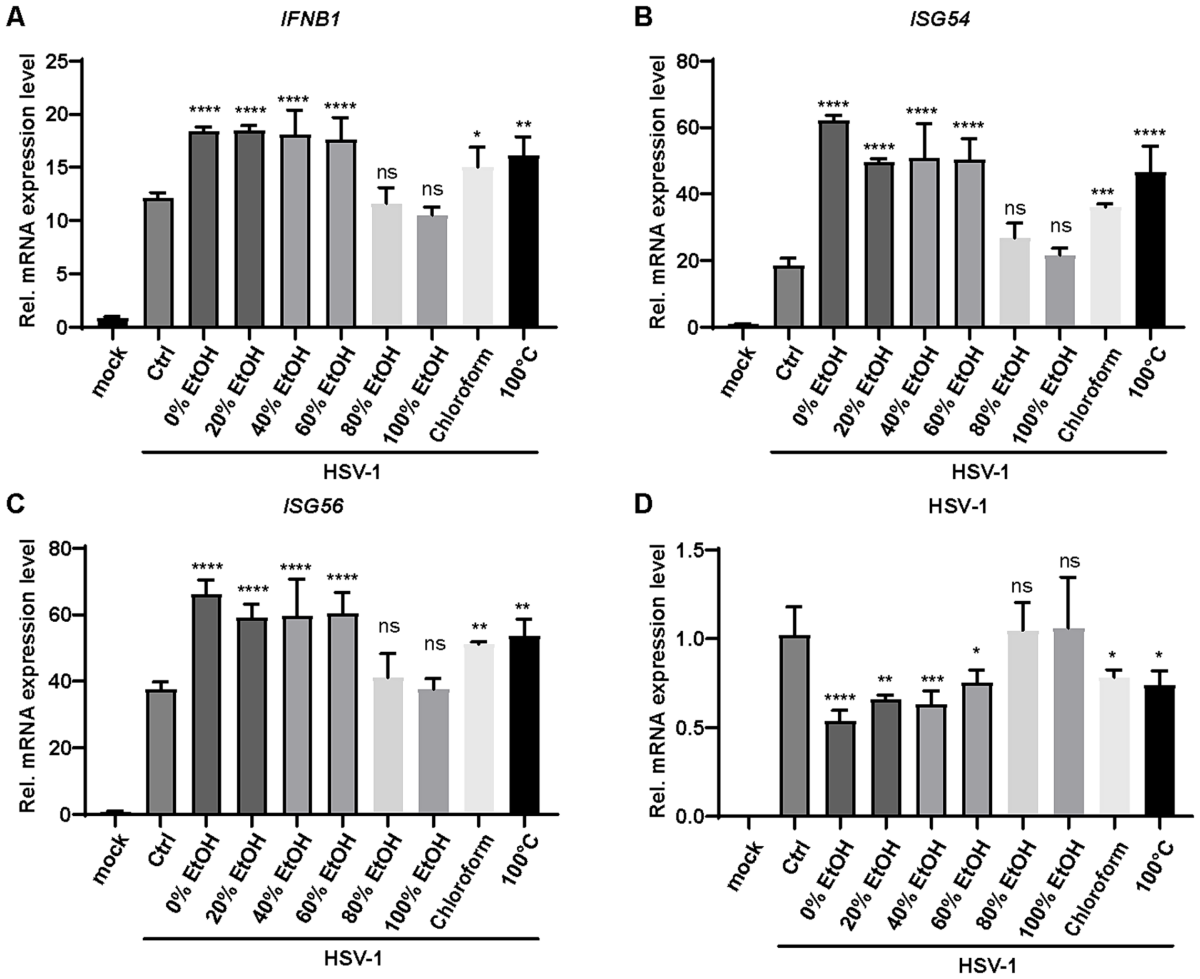
Regulation of the IFN-I antiviral signaling pathway by various components of metabolites derived from *E. coli* JNL*-EC1*. **(A–D)** THP-1 cells were pretreated for 2 h with 10 μL of lyophilized *E. coli* JNL*-EC1* metabolites reconstituted in varying concentrations of ethanol, chloroform, or heat-treated (100 °C for 15 min) bacterial culture supernatant, followed by infection with HSV-1. At 24 h postinfection, cells were harvested, and qPCR analysis was performed to measure the mRNA levels of *IFNB1* **(A)**, *ISG54* **(B)**, *ISG56* **(C)**, and *HSV-1* **(D)**. Each group was compared with the Ctrl group. ns, not significant; *p < 0.05, **p < 0.01, ***p < 0.001, and ****p < 0.0001; Student’s *t*-test, *n* = 4.

To further characterize the active components within the metabolites of *E. coli JNL-EC1* that modulate antiviral immune responses, we used graded concentrations of ethanol and chloroform to extract distinct fractions from lyophilized *E. coli JNL-EC1* metabolites. Treatment with the fraction soluble in 0–60% ethanol significantly enhanced the expression of *IFNB1*, *ISG54*, and *ISG56* following HSV-1 infection (Figures 7A–C) and suppressed viral replication (Figure 7D). In contrast, the fraction dissolved by ethanol concentrations above 60% lacked these activities, suggesting that the active components preferentially dissolve in water and are highly polar. Notably, the chloroform-extracted fraction also promoted *IFNB1*, *ISG54*, and *ISG56* expression and inhibited HSV-1 replication, indicating that certain active components are nonpolar and lipid-soluble. These two distinct solubility profiles suggest the presence of more than one active ingredient. Moreover, heat-treated *E. coli JNL-EC1* metabolites largely retained their ability to upregulate *IFNB1*, *ISG54*, and *ISG56* expression and suppress HSV-1 replication, demonstrating that the majority of the active components are heat-stable and unlikely to be proteins (Figure 7).

### 3.8 *Escherichia coli JNL-EC1* suppresses DNA and RNA virus replication *in vitro*

**Figure 8:**
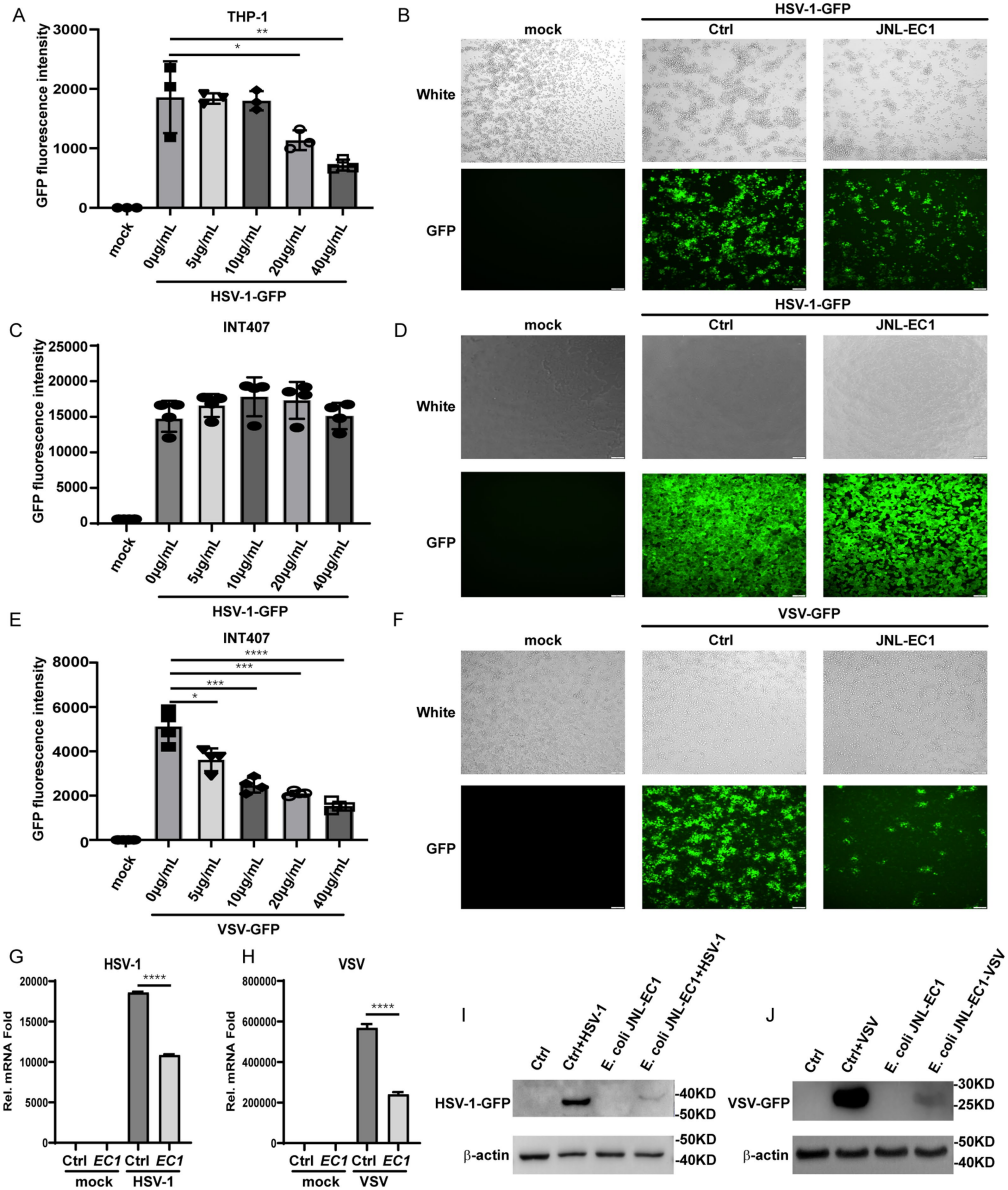
*Escherichia coli* JNL*-EC1* enhances cGAS/STING and RNA virus induced antiviral response. THP-1 macrophages and INT407 epithelial cells pretreated with 20 μg/mL JNL-EC1 metabolites for 2 h were infected with: GFP-tagged HSV-1/GFP-tagged VSV. Antiviral effects assessed at 24 hpi by: fluorescence intensity **(A,C,E)**, fluorescence microscope **(B,D,F)**, qPCR assay **(G,H)**, and western blot **(I,J)**. ns, Not significant; *p < 0.05, **p < 0.01, ***p < 0.001, and ****p < 0.0001; Student’s *t*-test, *n* ≥ 3.

Given that metabolites derived from *E. coli JNL-EC1* enhance IFN-I induction following DNA and RNA virus infection, we next investigated their functional impact on antiviral immunity. Viral replication was assessed by monitoring GFP fluorescence intensity in GFP-tagged herpes simplex virus 1 (HSV-1) and vesicular stomatitis virus (VSV). Pretreatment with *E. coli JNL-EC1* metabolites significantly reduced HSV-1–associated GFP signals in THP-1 cells, which harbor an intact cGAS-STING signaling axis, but not in INT407 cells, which are deficient in functional cGAS-STING pathway components (Figures 8A–D). These findings suggest that the antiviral effect against HSV-1 is mediated through potentiation of the cGAS-STING pathway. Notably, *E. coli JNL-EC1* metabolites also suppressed VSV-derived GFP fluorescence in INT407 cells (Figures 8E,F), indicating additional pathway engagement. Consistent with these observations, qPCR and Western blot analyses revealed that metabolite pretreatment reduced both HSV-1 and VSV mRNA levels and diminished GFP protein expression (Figures 8G–J). Collectively, our results demonstrate that metabolites from *E. coli JNL-EC1* markedly inhibit replication of both DNA and RNA viruses.

### 3.9 *Escherichia coli JNL-EC1* enhances antiviral immunity in a mouse model of viral infection

**Figure 9:**
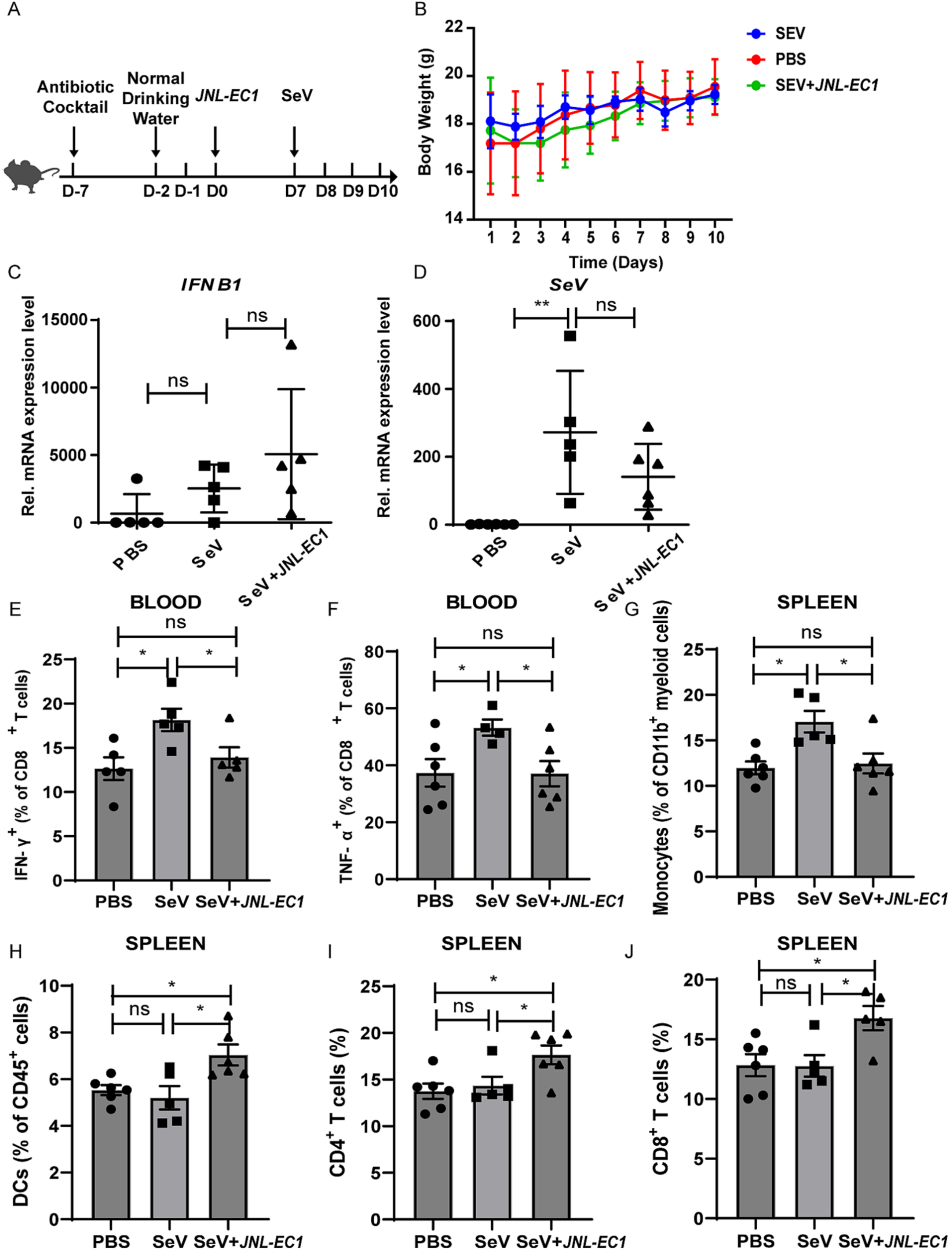
*Escherichia coli JNL-EC1* promotes antiviral immunity *in vivo*. **(A)** Experimental protocol of testing *E. coli JNL-EC1* in SeV-infected mice. **(B)** Body weight was monitored for 10 days after gastric gavaged with *E. coli JNL-EC1*. **(C,D)** The mRNA levels of *IFNB1* and SeV in spleen mononuclear cells were analysis by qPCR assay. **(E,F)** IFNγ/TNFα expression of CD8 + T cells in blood were analysis by flow cytometry. **(G–J)** The monocytes, dendritic cell (DCs), CD4 + T cells and CD8 + T cells in spleen were analysis by flow cytometry. ns, not significant; *p < 0.05 and **p < 0.01; One-way ANOVA, *n* ≥ 5.

Having established that metabolites from *E. coli JNL-EC1* inhibit DNA and RNA virus replication *in vitro*, we next investigated their impact on antiviral cellular immunity *in vivo* using a SeV-infected mouse model (Figure 9A). No overt differences in morphology, behavior, or weight gain were observed following SeV infection or intragastric administration of the *E. coli JNL-EC1* (Figure 9B). Treatment with *E. coli JNL-EC1* upregulated *Ifnb1* mRNA expression in splenic mononuclear cells following SeV infection (Figure 9C). Concurrently, *E. coli JNL-EC1* pre-treatment led to a reduction in SeV mRNA levels, although these changes did not achieve statistical significance (Figure 9D). Innate immunity constitutes the primary defense against viral pathogens. Given the observed enhancement of IFN-I-mediated antiviral responses by *E. coli JNL-EC1*, we assessed its influence on immune cell function in vivo. Pre-treatment with *E. coli JNL-EC1* attenuated IFN-*γ* and TNF-*α* expression in circulating CD8^+^ T cells (Figures 9E,F) and reduced the frequency of monocytes in the spleen following SeV infection (Figure 9G). Conversely, intragastric administration of the *E. coli JNL-EC1* increased the proportions of dendritic cells (DCs), CD4^+^ T cells, and CD8^+^ T cells in the spleen (Figures 9H–J). Collectively, these results demonstrate that intestinal *E. coli JNL-EC1* modulate systemic immune cell populations during viral challenge, highlighting a role for the gut microbiota in shaping host antiviral immunity.

## 4 Discussion

As the mechanisms underlying the interplay between the gut microbiota and host immunity become increasingly well-defined, the role of gut bacteria in modulating immune responses has gained broader recognition (Pan et al., 2023). Infants are particularly vulnerable to viral infections due to the immaturity of their immune system, particularly the adaptive branch. Moreover, the limited metabolic capacity of the liver and kidneys in early life restricts the use of conventional antiviral drugs, which often carry significant safety concerns and risks in this population. Currently, specific gut commensals, such as *Bifidobacteria* species, have been shown to play critical roles in regulating immune system development during early infancy (Henrick et al., 2021; Donald and Finlay, 2023; Lin et al., 2022). Probiotic administration in infants offers advantages over traditional antiviral therapies, including higher acceptability and fewer adverse effects. Therefore, modulating host antiviral immunity through gut microbiota intervention may represent a promising strategy for addressing viral infections in infants.

It is now well established that the commensal microbiota modulates a broad spectrum of disease processes (Pan et al., 2023; Ruff et al., 2020; Stacy et al., 2021; Choi et al., 2021). The advent of next-generation sequencing has enabled comprehensive profiling of the associations between gut microbiome composition and disease progression. Although 16S rRNA gene amplicon and metagenomic sequencing have facilitated the identification of differentially abundant bacterial taxa linked to various disease states, the functional contributions of these microbes to disease modulation remain largely unexplored. To establish causal relationships between specific intestinal bacteria and host biological processes, we isolated individual bacterial strains and functionally characterized their immunomodulatory or regulatory activities using *in vitro* systems.

The human gastrointestinal tract hosts a complex microbial ecosystem comprising bacteria, fungi, protozoa, archaea, and viruses. This gut microbiota plays an essential role in host defense, both by conferring colonization resistance against pathogenic microorganisms (Perez-Lopez et al., 2016; Zou et al., 2021) and by priming IFN-I responses to bolster antiviral immunity (Stefan et al., 2020; Winkler et al., 2020; Erttmann et al., 2022; Bradley et al., 2019; Wirusanti et al., 2022). To systematically investigate these immunomodulatory functions, we characterized 45 bacterial strains derived from the human gut for their capacity to modulate IFN-I expression. Our findings reveal distinct functional clusters: strains of *Escherichia* and *Leuconostoc* significantly potentiate *IFNB1* expression following SeV infection, whereas *Lactiplantibacillus* strains exhibit inhibitory effects. This observation presents a notable departure from conventional microbiological paradigms, wherein *Escherichia* species are typically regarded as opportunistic pathogens, while *Lactiplantibacillus* are widely recognized as beneficial probiotics. These results challenge the traditional pathogen-probiotic dichotomy and suggest that certain “pathogenic” gut microbes may paradoxically enhance host antiviral defenses via modulation of the IFN-I pathway. This underscores the necessity for functional-rather than purely taxonomic-characterization of microbiota-host interactions.

In this study, we found that *E. coli JNL-EC1* promotes IFN-I expression following both RNA virus and DNA virus infection, thereby enhancing antiviral innate immunity and exerting protective antiviral effects. Further analysis of its active components revealed that the metabolites responsible for virus-induced IFN-I expression are likely not a single entity but rather a combination of multiple components. These active metabolites exhibit greater solubility in water than in ethanol, suggesting strong hydrophilicity. However, when we extracted the metabolites using chloroform, the active components were also present in the organic phase. These two distinct solubility profiles suggest the presence of more than one active ingredient. Additionally, the active components appear to possess a certain degree of heat tolerance, making it unlikely that they are proteins (Figure 7). Future studies aimed at identifying these active components will provide valuable guidance for the development of antiviral immunotherapeutics and probiotic-based interventions.

Our findings demonstrate that metabolites from *E. coli JNL-EC1* enhance IFN-I-mediated antiviral responses *in vitro*. Although gastric gavage with live *E. coli JNL-EC1* increased *IFNB1* mRNA expression and reduced SeV infection *in vivo*, these effects did not reach statistical significance, likely due to inter-individual variability among mice and the limited sample size. Further studies are warranted to evaluate the potential of *E. coli JNL-EC1* as an adjunctive probiotic therapy against viral infections.

## Data Availability

*Escherichia coli JNL-EC1* Whole Genome Shotgun project has been deposited at DDBJ/ENA/GenBank under the accession JBPAPQ000000000. The version described in this paper is version JBPAPQ010000000.

## Footnotes

1
https://automlst2.ziemertlab.com/

2
https://www.chiplot.online/

3
http://www.mgc.ac.cn/VFs/
