# Supplementary material for: Escherichia coli JNL-EC1 enhances type I IFN-mediated antiviral response during DNA and RNA virus infection
Source: Front Microbiol. 2026 Jun 3;17:1820773. doi: 10.3389/fmicb.2026.1820773 (PMC13272492; doi:10.3389/fmicb.2026.1820773)
Supplement: Supplementary file 2 [file Table_2.DOCX]

| **Deposit Number** | **Scientific Name** |
| --- | --- |
| 1 | *Bifidobacterium longum subsp 1* |
| 2 | *Bifidobacterium breve 1* |
| 3 | *Bifidobacterium animalis subsp 1* |
| 4 | *Salinicoccus salitudinis* |
| 5 | *Escherichia fergusonii* |
| 6 | *Bifidobacterium bifidum 1* |
| 7 | *Lactococcus lactis 1* |
| 8 | *Bifidobacterium animalis subsp 2* |
| 9 | *Bifidobacterium longum subsp 2* |
| 10 | *Bifidobacterium longum subsp 3* |
| 11 | *Enterococcus avium 1* |
| 12 | *Enterococcus sp* |
| 13 | *Bifidobacterium breve 2* |
| 14 | *Cronobacter sakazakii* |
| 15 | *Enterococcus avium 2* |
| 16 | *Enterococcus durans* |
| 17 | *Bifidobacterium breve 3* |
| 18 | *Bifidobacterium pseudocatenulatum 1* |
| 19 | *Streptococcus sp.* |
| 20 | *Lactococcus garvieae 1* |
| 21 | *Ligilactobacillus saerimneri* |
| 22 | *Streptococcus macedonicus* |
| 23 | *Enterococcus avium 3* |
| 24 | *Lactiplantibacillus plantarum 1* |
| 25 | *Bifidobacterium longum subsp 4* |
| 26 | *Leuconostoc lactis 1* |
| 27 | *Escherichia coli* |
| 28 | *Enterococcus italicus* |
| 29 | *Streptococcus pasteurianus* |
| 30 | *Leuconostoc lactis 2* |
| 31 | *Weissella cibaria* |
| 32 | *Bifidobacterium pseudocatenulatum 2* |
| 33 | *Bifidobacterium longum subsp 5* |
| 34 | *Lactococcus garvieae 2* |
| 35 | *Lacticaseibacillus paracasei* |
| 36 | *Lactococcus lactis 2* |
| 37 | *Lactobacillus sp* |
| 38 | *Bifidobacterium bifidum 2* |
| 39 | *Bifidobacterium pseudocatenulatum 3* |
| 40 | *Bifidobacterium breve 4* |
| 41 | *Enterococcus faecium* |
| 42 | *Lactiplantibacillus plantarum 2* |
| 43 | *Bifidobacterium pseudocatenulatum 4* |
| 44 | *Bifidobacterium breve 5* |
| 45 | *Enterococcus hirae* |
